# Towards Automated Quality Assurance: Integrating Deep Learning and Classical ML into the Digital Radiography Pipeline

**DOI:** 10.3390/diagnostics16132111

**Published:** 2026-07-06

**Authors:** Hsuan-Yu Chen, Cheng-Fu Chou, Sheng-Hung Liao, Meng-Hsun Wu, Kuan-Yi Chen, Ta-Wei Yang, Jungwei Wilfred Fan, Chih-Hao Chang

**Affiliations:** 1 Department of Orthopedic Surgery, College of Medicine, National Taiwan University, No.7, Chung-Shan South Road, Zhong-Zheng District, Taipei 100, Taiwan; hychen83@ntu.edu.tw; 2 Department of Orthopedic Surgery, Mayo Clinic, Rochester 55905, MN, USA; 3 Graduate Institute of Networking and Multimedia, National Taiwan University, Taipei 106, Taiwan; ccf@csie.ntu.edu.tw (C.-F.C.); dwyang@cmlab.csie.ntu.edu.tw (T.-W.Y.); 4 Department of Computer Science and Information Engineering, National Taiwan University, Taipei 106, Taiwan; r10922135@ntu.edu.tw (S.-H.L.); d10922001@ntu.edu.tw (M.-H.W.); r12922100@ntu.edu.tw (K.-Y.C.); 5 Department of Artificial Intelligence and Informatics, Mayo Clinic, Rochester 55905, MN, USA; fan.jung-wei@mayo.edu

**Keywords:** lumbar spine X-ray, quality control, deep learning, automated, patient safety

## Abstract

**Background/Objectives:** To develop and evaluate a deep learning-based quality control system for Lumbar Spinal Digital Radiographs (LSDR), designed to automate and improve their evaluation and reduce reliance on manual reviews. **Methods:** This retrospective study utilized a deep learning workflow comprising image segmentation, feature extraction, and a classification model. The dataset, including anteroposterior (AP) and lateral (LAT) X-ray images, was expanded through data augmentation techniques. Four U-Net-based models were assessed: standard U-Net, Swin-UNet, Attention U-Net, and Attention U-Net with the weight map, with the latter selected for its superior performance. Extracted features, such as brightness, contrast, and anatomical positioning, were used in an XGBoost classifier, which was evaluated using mean intersection over union (mIoU), accuracy, sensitivity, specificity, and AUC. **Results:** The Attention U-Net with weighted attention outperformed the other models, achieving high mIoU scores in both AP and LAT views. The XGBoost classifier achieved the best performance in classifying images as “qualified” or “unqualified,” with an AUC of approximately 0.9, high accuracy, and balanced sensitivity and specificity. This approach effectively addressed class imbalances and improved model accuracy compared to traditional machine learning models such as MLP and SVM. **Conclusions:** The developed automated quality control system demonstrated potential for enhancing image quality, enhancing diagnostic reliability, and optimizing clinical workflow efficiency.

## 1. Introduction

Lumbar spinal digital radiograph (LSDR) imaging is a fundamental tool in clinical practice and serves as the first-line diagnostic tool before advanced modalities are employed [1]. It provides valuable insight into the structure and condition of the spine, enabling the early detection and evaluation of various disorders, such as abnormal spinal curvature, degenerative changes, fracture, spondylolisthesis, osteoporosis, tumors or infection [2,3]. At large medical centers, LSDRs are performed in high volumes; for example, at the National Taiwan University Hospital, over 30,000 LSDR are produced annually. Ensuring optimal image quality is essential for accurate diagnoses and treatment planning. Approximately 9% of DR are rejected because of factors such as patient mispositioning, improper exposure, and technical issues [4]. These quality issues lead to repeated imaging, increased patient radiation exposure, and increased healthcare costs [5].

In recent years, deep learning has emerged as a transformative technology in the medical field, particularly in the domain of medical imaging [6,7]. Deep learning algorithms have demonstrated remarkable success in tasks such as image recognition, classification, and segmentation, making them well suited for applications in diagnostic imaging. For example, deep learning models have been used to detect brain lesions in MRI scans, aiding in the early detection of neurological disorders [8]. Similarly, deep learning techniques have been employed to segment and classify polyps in colonoscopy images [9] and detect breast tumors in mammograms [10]. These advancements illustrate how deep learning can be leveraged to assist physicians in making accurate and timely diagnoses, ultimately improving patient outcomes. Advancements in medical imaging technology and the integration of artificial intelligence have further enhanced the diagnostic capabilities of LSDR [11,12,13,14]. While deep learning has demonstrated success in medical imaging tasks, there remains a need to fully integrate these technologies into automated quality control (QC) for LSDR.

The primary objective of the study was to develop an automated system that evaluates whether LSDR images meet established diagnostic quality standards. This system incorporates domain knowledge from medical experts to inform the segmentation and feature extraction processes. A machine learning classifier, specifically trained to distinguish between qualified and unqualified images, ensures that the automated evaluations meet professional standards. This system aims to support radiographers in producing high-quality diagnostic images, thereby reducing the workload and potential for human error associated with manual reviews. Given these challenges, we suggest that automating the QC process can significantly improve efficiency and accuracy. This study proposes a deep-learning-based QC system designed to automate and enhance plain film evaluations, thereby reducing the dependency on manual assessments in the clinical workflow.

## 2. Methods

**Figure 1:**
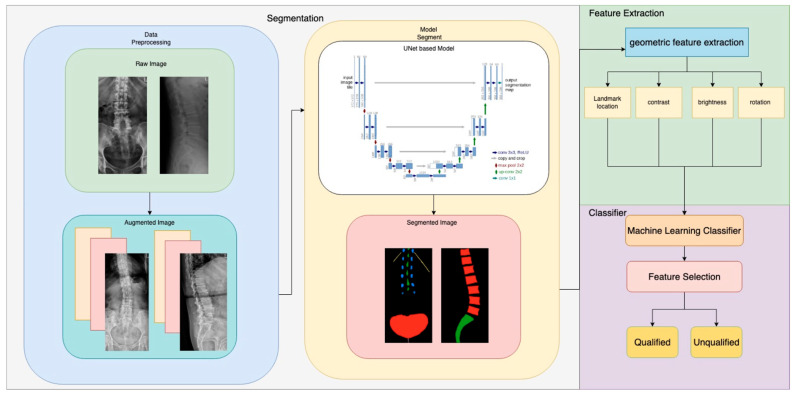
Workflow of the study.

In this section, we present the methodology adopted to develop an automated QC system for LSDR images. The process is divided into three main stages. First, we describe the data preprocessing pipeline, including annotation, augmentation, and preparation steps to ensure the dataset is suitable for model training. Second, we introduce the segmentation stage, where a deep learning-based model is employed to accurately identify key anatomical structures in the LSDR images. Finally, we detail the construction of the QC model, in which geometric features extracted from the segmented images are combined with machine learning classifiers to distinguish between qualified and unqualified images. Figure 1 illustrates the workflow of this study. This study was conducted with the approval of the Institutional Review Board (IRB) of National Taiwan University Hospital under protocol number 202107102RIND.

### 2.1. Datasets

All imaging data utilized in this study were retrospectively collected from the database at National Taiwan University Hospital (NTUH), and initial raw dataset comprised approximately 580,000 both anteroposterior (AP) and lateral (LAT) LSDR images. A preliminary convolutional neural network (CNN) classifier was trained on a representative subset of 2000 expert-verified images, and the model was deployed to organize the entire database into distinct AP and LAT cohorts for downstream processing.

**Figure 2:**
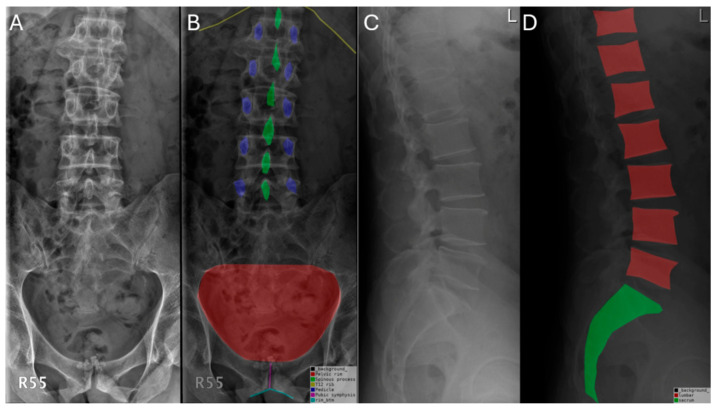
Landmark annotation on anterior–posterior (AP) and lateral (Lat) plain radiographs. (**A**) Original AP radiograph; (**B**) AP radiograph with annotated landmarks: spinous process (green), pedicle (blue), T12 rib (yellow), pubic rim (red), pubic symphysis (pink), and inferior margin of the pubic rim (light blue); (**C**) Original LAT radiograph. (**D**) LAT radiograph with annotated vertebral bodies from T11 to L5 (red) and sacrum (green).

The first part of the dataset consists of medical LSDR images in which we manually annotated key anatomical landmarks in AP and LAT views. These landmarks can be used to identify the lumbar region and support subsequent feature extraction, and the annotation process was performed using LabelMe, an open-source Python-based tool for image labeling. Anatomical landmarks were manually annotated on AP and LAT plain radiographs to provide reference points for model training and evaluation (Figure 2). Landmark annotation was independently performed by one orthopedic surgeon and one radiologist. A senior radiologist was consulted in case of disagreement, and a final consensus was reached through joint review. For the AP view, annotations included the spinous process (green), pedicle (blue), T12 rib (yellow), pubic rim (red), pubic symphysis (pink), and inferior margin of the pubic rim (light blue). For the LAT view, the vertebral bodies from T11 to L5 were labeled in red, and the sacrum was labeled in green. After completing the labeling process, we obtained 360 AP and LAT view images annotated images that were used for the image segmentation task.

**Figure 3:**
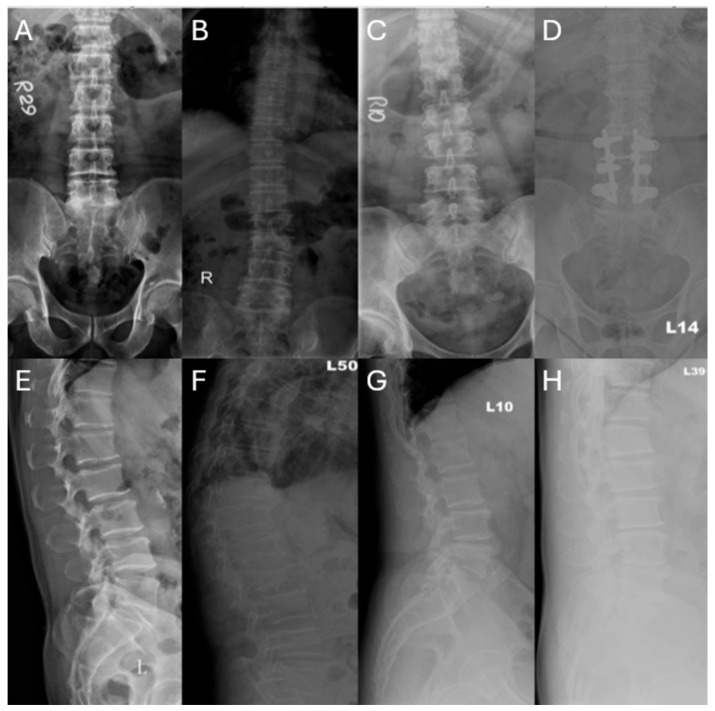
Definition of image quality on AP and Lat plain radiographs. (**A**) Standard AP radiograph meeting all quality criteria. (**B**) AP radiograph with incomplete coverage of the pelvic brim. (**C**) AP radiograph with off-center positioning. (**D**) AP radiograph with overexposure. (**E**) Standard Lat radiograph of the lumbosacral spine meeting all quality criteria. (**F**) Lat radiograph with incomplete sacral coverage. (**G**) Lat radiograph with incomplete T11 coverage. (**H**) Lat radiograph with overexposure.

In addition to the segmentation dataset, we also constructed a dataset for binary classification. The second portion of the dataset includes 540 AP view and 540 LAT view LSDR images evaluated for quality using binary classification (denoted as “qualified” or “unqualified”) based on medical guidelines. To ensure a balanced evaluation, the distribution of image quality within this classification dataset contained 50% qualified and 50% unqualified images. The criteria included image centering, exposure levels, lumbar region coverage, and patient positioning. Image quality was evaluated based on predefined radiographic criteria for both AP and LAT views. For the AP view, suboptimal images included those with incomplete coverage of the pelvic brim, off-center positioning, or overexposure. For the LAT view, suboptimal images included those with incomplete coverage of the sacrum or T11 vertebra, or with overexposure. Representative examples of each category are shown in Figure 3.

### 2.2. Image Segmentation Model

**Figure 4:**
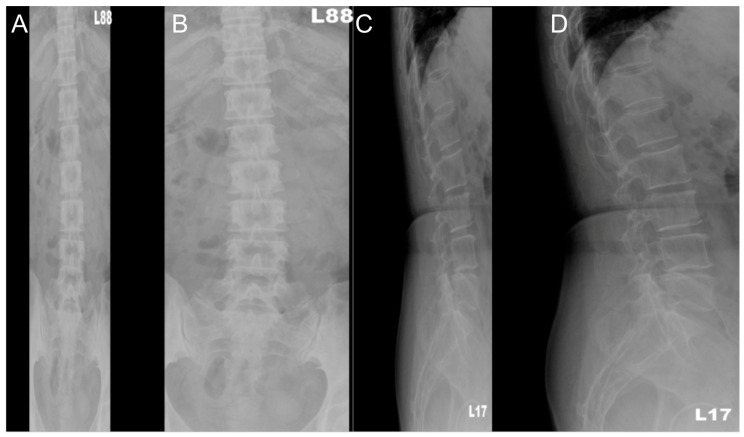
Effect of image cropping on AP and LAT plain radiographs. (**A**,**C**) Original images before cropping. (**B**,**D**) The same images after proper cropping, which removes irrelevant background while preserving all critical anatomic structures. Care must be taken to avoid over-cropping, which can remove diagnostically important regions.

Before building the segmentation model, several data preprocessing steps were applied to ensure that the images were clean and standardized. We cropped the images and removed unnecessary black borders, which helped normalize the input size and eliminate irrelevant background regions, as shown in Figure 4. Additionally, data augmentation, including flipping, histogram equalization, and random rotation, was adopted to expand the dataset from 360 to 1440 images, enhancing variability and model robustness.

In the segmentation stage, four U-Net-based models were adopted and evaluated: the standard U-Net, Swin-UNet, Attention U-Net, and Attention U-Net with the weight map. U-Net, known for its “U”-shaped encoder–decoder structure with skip connections, enables accurate pixel-level segmentation. Swin-UNet replaces CNNs with Swin Transformer modules, whereas Attention U-Net includes attention gates to enhance the focus on key regions. To address the data imbalance issue, we proposed a weighted cross-entropy loss that incorporates a modified version of the FU-Net weight map [15] function. The loss function is designed as a weighted cross-entropy:

$$Loss=\sum_{x=\Omega }w\left(x\right)\log\left(p_{l\left(x\right)}(x)\right)$$

Here, $w\left(x\right)$ is the weighted map function. Our proposed weighted map function can be expressed as follows:

$$w\left(x\right)=\frac{1}{w_{c}\left(x\right)}\times e^{-log100\times p_{l}{\left(x\right)}^{\beta }}$$

$$w_{c}\left(x\right)=\frac{x}{x_{min}}\;,\;$$

$$x_{min}=min(x_{0},x_{1},\ldots ,x_{k})$$

where k represents the number of label classes, $x_{min}$ indicates the class that occupies the smallest pixel area in the image, and $x_{k}$ denotes the pixel count for class k. Therefore, $w_{c}\left(x\right)\;$ is the ratio of the other classes relative to the class with the smallest number. Additionally, we use the hyperparameter β=3. While the original FU-Net function naturally directs the model to focus more on error-predicted pixels, our modification serves to mitigate class imbalance and accounts for the distance between cells. The weighted map assigns greater emphasis to the least frequent class in the image, ensuring that it receives more attention during the training process.

Our proposed weight map effectively handles class imbalances, particularly concerning the T12 rib in the AP view, and emphasizes incorrectly predicted areas during training. By focusing on these aspects, we aimed to improve the accuracy and robustness of the model for identifying key landmarks in lumbar spine images.

We used 5-fold cross-validation for robust evaluation. Specifically, 80% of the data were used for training, and 20% were used for testing, with a training split of 4:1 for cross-validation. All augmentation operations were performed on the training partition within each cross-validation fold, and the test set in each fold consisted exclusively of original, non-augmented images to prevent data leakage. Model performance was evaluated using the mean intersection over union (mIoU), which measures segmentation accuracy by assessing the overlap between predicted and actual regions. The mIoU averages the IoU scores across all classes, thereby comprehensively evaluating model performance.

### 2.3. Feature Extraction from the LSDR Images

After segmentation, geometric features were extracted from the images to incorporate medical domain knowledge into the analysis, such as landmark positions, brightness and contrast levels, and lumbar rotation. Using the breadth-first search algorithm, we identified landmarks and calculated the centroids for precise positioning. In the AP view, we focused on the lumbar spine and pelvic rim to ensure accurate representation. We also marked the presence of other landmarks, such as the T12 rib and pubic symphysis. In the LAT view, we confirmed the presence of the T11, T12, and lumbar vertebrae without exact coordinates. The inclusion of the sacrum was ensured by a pixel count to verify diagnostic adequacy. Next, we measured the average brightness and its variance for uniform light distribution, which is crucial for clear anatomical visibility. To enhance precision, we used a lumbar brightness contrast metric calculated using the Weber contrast equation. It is defined as

$$C=\frac{L_{Lumbar}-L_{Background}}{L_{Background}}$$

$L_{Lumbar}$ represents the average brightness value measured within the lumbar region and $L_{Background}$ represents the average brightness value of the surrounding background area. The Weber contrast equation assesses the difference between the lumbar and background brightness, focusing on the pedicles for a consistent reference. The background brightness was averaged from the non-lumbar areas. This helps gauge the clarity of the lumbar region, which is crucial for high-quality LSDR imaging. For lumbar rotation, we used relative measurements by comparing the distances from the pedicles to the spinous process and estimating the rotation using a derived ratio.

$$Lumbar\;Rotation\;Ratio=\frac{\delta_{a}}{\delta_{b}}$$

where $\delta_{a}$ and $\delta_{b}$ are the distance between the left pedicle and spinous process and the distance between the right pedicle and spinous process, respectively. This approximation provides valuable insight into whether the patient was properly positioned during radiography, which is a critical factor for maintaining the quality and diagnostic value of the images.

### 2.4. Quality Control Classifier

After feature extraction, we applied a QC classifier using Extreme Gradient Boosting (XGBoost) [16] to determine whether the LSDR images met the diagnostic standards. XGBoost works by sequentially building decision trees and correcting errors to enhance accuracy, with built-in regularization to prevent overfitting. We chose XGBoost because of its known efficiency and ability to handle large datasets. Specifically, the task was to classify images as “qualified” or “unqualified” based on features such as landmark positions, brightness, contrast, and rotation. The model performance was assessed using a confusion matrix detailing true positives (TP), true negatives (TN), false positives (FP), and false negatives (FN). Metrics such as accuracy (ACC) measure the proportion of correctly classified images and provide insights into the classifier’s overall performance.

$$ACC=\frac{TP+TN}{TP+TN+FP+FN}$$

Sensitivity (SEN) is also known as the recall or true-positive rate. SEN evaluates the ability of the model to correctly classify unqualified images. It quantifies how well the model detects true positives, ensuring that unqualified images are correctly identified. This metric can be written as

$$SEN=\frac{TP}{TP+FN}$$

Specificity (SPE) measures the true negative rate, indicating the ability of the model to accurately classify qualified images. This reflects the proportion of qualified images that are correctly identified, helping to assess the model’s performance in rejecting unqualified predictions. SPE is defined as

$$SPE=\frac{TN}{TN+FP}$$

Balanced accuracy (BAC) is the average of sensitivity and specificity. It is particularly useful when dealing with imbalanced datasets, as it provides a more balanced evaluation by accounting for both classes equally, rather than favoring the more frequent class. BAC is defined as

$$BAC=\frac{SEN+SPE}{2}$$

Finally, the area under the curve (AUC) measures the model’s ability to distinguish between “qualified” and “unqualified” classes derived from the receiver operating characteristic (ROC) curve. AUC values closer to 1 indicate better classification performance. These evaluation metrics provide a comprehensive assessment of the classifier’s performance, capturing both its overall accuracy and its ability to correctly identify qualified and unqualified images.

## 3. Results

### 3.1. Segmentation

**Figure 5:**
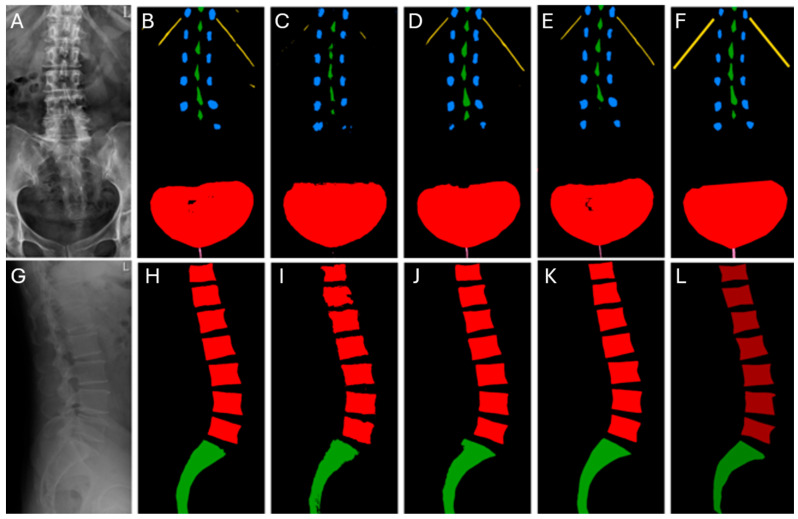
Examples of segmentation outputs on AP and Lat plain radiographs. (**A**) Original AP radiograph. (**B**–**E**) Segmentation results from U-Net, Swin U-Net, Attention U-Net, and Attention U-Net with weighted map, respectively. (**F**) Ground truth segmentation for the AP view. (**G**) Original Lat radiograph. (**H**–**K**) Segmentation results from U-Net, Swin U-Net, Attention U-Net, and Attention U-Net with weighted map, respectively. (**L**) Ground truth segmentation for the Lat view.

In the AP view, as shown in Table 1 and illustrated in Figure 5A–F, the Attention U-Net with the weight map (Figure 5E) outperformed all other models, achieving the highest mean IoU of 0.8317 compared with 0.8203 for the original Attention U-Net (Figure 5D), 0.8157 for U-Net (Figure 5B), and 0.7540 for Swin U-Net (Figure 5C). In addition, in the LAT view, as reported in Table 1 and shown in Figure 5G–L, the Attention U-Net with the weight map (Figure 5K) again reached the best score, with a mean IoU of 0.9146, surpassing the original Attention U-Net (Figure 5J) at 0.8913, U-Net at 0.8835 (Figure 5H), and Swin U-Net at 0.8413 (Figure 5I). The higher mIoU observed in the LAT view is due to the relative simplicity of the labeling task, since the anatomical structures in the lateral perspective are more regular in shape and easier to identify compared with the AP view. The relatively poor performance of Swin U-Net can be attributed to overfitting, as transformer-based architecture typically requires much larger datasets to generalize effectively, whereas our dataset size was limited.

These results demonstrate that the attention mechanism effectively directs the model to focus on relevant anatomical regions, while the weight map further enhances performance by addressing class imbalance and forcing the network to refine mis predicted pixels, resulting in more accurate segmentation boundaries. Therefore, the Attention U-Net with the weight map was selected as the final segmentation model for subsequent feature extraction and QC tasks.

### 3.2. Performance of Quality Control Classifier

In evaluating different classifiers for the QC task, several approaches were considered. The quality control performance of each model is shown in Table 2 (AP view) and Table 3 (LAT view). A multilayer perceptron (MLP) served as a simple feedforward neural network capable of learning nonlinear mappings between features and labels, while a self-attention classifier leveraged attention mechanisms to highlight the most informative features and capture their dependencies. Traditional machine learning methods were also included. Support vector machines (SVM) sought an optimal hyperplane to separate qualified and unqualified images, and random forests combined multiple decision trees to improve robustness and reduce overfitting. Extreme Gradient Boosting (XGBoost) was employed as a powerful ensemble method that builds trees sequentially with regularization, offering both efficiency and strong performance on structured data.

**Figure 6:**
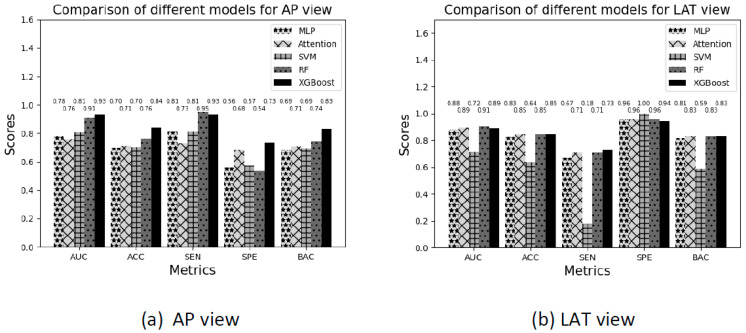
Comparison of different models for quality control.

As presented in Figure 6a, XGBoost achieved the best overall performance in AP view among all classifiers, with an AUC of 0.9328, ACC of 0.8406, SEN of 0.9300, SPE of 0.7317, and BAC of 0.8309. Compared with MLP, self-attention, SVM, and random forest, XGBoost provided a more stable balance between sensitivity and specificity. It effectively identified unqualified images with a high SEN and maintained a relatively high SPE. Random forest achieved a competitive AUC but had lower specificity. MLP and the self-attention classifier showed lower overall performance, whereas SVM yielded moderate results but struggled with specificity.

For the LAT view, Figure 6b reveals that XGBoost achieved the best balanced performance, with an AUC of 0.8906, accuracy of 0.8468, sensitivity of 0.7272, specificity of 0.9420, and balanced accuracy of 0.8347, closely followed by the self-attention classifier and Random Forest. Although Random Forest attained a slightly higher AUC (0.9071), XGBoost maintained a more favorable sensitivity-specificity trade-off, which is more meaningful for flagging unqualified images. SVM performed poorly in terms of sensitivity, making it less suitable for detecting unqualified images. These findings confirm the superior capability of XGBoost to classify LSDR images, which is attributed to its effective handling of complex data relationships and built-in regularization that prevents overfitting.

Overall, the comparative results across both AP and LAT views consistently demonstrate that XGBoost offers the most reliable and clinically meaningful performance among the evaluated classifiers.

## 4. Discussion and Limitations

### 4.1. Discussion

Traditional LSDR image QC faces multiple challenges related to several variations, including inconsistent centering and coverage, exposure parameter variability with manual selection of kilovoltage (kVp) and milliamperage-seconds (mAs) of over- or underexposure, technologist experience, and equipment calibration. Image QC assessment remains largely dependent on manual visual inspection by technologists or radiologists after image acquisition in current clinical workflows. Poor QC images related to patient rotation, field clipping, or suboptimal exposure are typically identified only in the post-acquisition stage, necessitating repeat imaging and resulting workflow inefficiencies. Feedback mechanism for early detection or flagging of substandard images before their transfer to the Picture Archiving and Communication System (PACS) may help on corrective action and decrease both patient radiation exposure and clinical turnaround time. To address these challenges, healthcare institutions have developed a set of medical image QC standard criteria aiming to reduce the number of poor-quality images and ensure that LSDR images consistently provide high diagnostic value. These standard criteria have been formulated through extensive clinical studies, combining both local insights and international best practices [17,18].

Deep learning is increasingly utilized in medical image QC and several studies have highlighted its potential for automating and improving QC processes. Automated image QC can streamline workflows, reduce errors, and reduce the number of retakes, allowing physicians to focus more on patient care. Chen et al. [19] proposed a deep-learning-based image QC framework to standardize radiography procedures. This framework segments and evaluates LSDR against predefined quality standards, automatically identifying key anatomical features to ensure proper exposure, positioning, and overall quality. This approach enhances consistency, accuracy, and efficiency in radiographic evaluations, thereby improving diagnostic reliability. Nousiainen et al. developed CNN models to assess three key quality criteria in PA chest radiographs based on prior QC efforts: lung inclusion, patient rotation, and appropriate inspiration [20]. An image annotation tool was created for visual assessment, and the model’s decision confidence was compared with human interobserver agreement to ensure accurate evaluations. Von Berg et al. [21] developed a method to automatically assess chest PA radiograph quality by evaluating the collimation, patient rotation, and inhalation state. Using three CNNs and two probabilistic anatomical atlases, key features were localized, and quality metrics were calculated according to international standards. Automated assessments were performed on a CPU in less than a second, demonstrating accuracy and robustness. This approach can help radiology departments maintain consistent image quality while reducing the risk of misdiagnosis and ensuring legal compliance.

**Figure 7:**
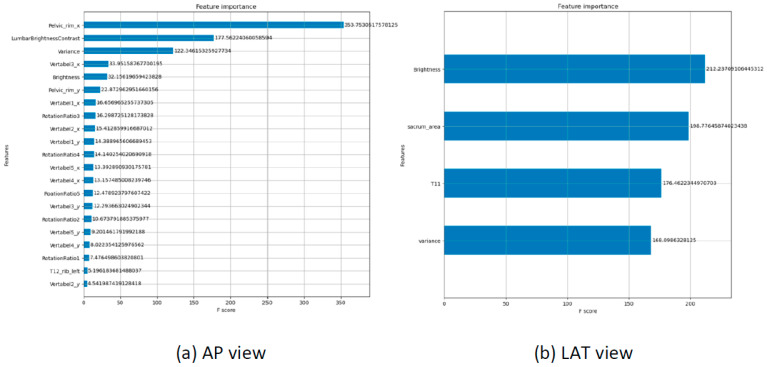
Feature importance for different views.

The architectural choice of a segmentation-first pipeline over a direct end-to-end deep learning classifier was driven by the critical need for clinical interpretability, and essential quality criteria, such as centering, anatomical coverage, exposure uniformity, and rotation, cannot be robustly assessed from raw images alone. These criteria require the precise localization of specific anatomical landmarks, including the pelvic rim, T12 rib, pubic symphysis, sacrum, and vertebral bodies, to accurately quantify their geometric relationships. Furthermore, we systematically evaluated over 20 candidate geometric features derived from the segmentation outputs during the feature selection phase. Figure 7a shows that in the AP view, XGBoost identified the pelvic rim x-coordinate as the most crucial feature, highlighting its importance for alignment. Lumbar brightness contrast is the second key feature for clear visualization, followed by variance, which indicates the image differentiation of anatomical structures. The XGBoost feature importance analysis for the LAT view in Figure 7b highlights overall brightness as the top feature, underscoring the importance of proper exposure. The sacrum is the second most significant feature and is crucial for spinal assessment. T11 ranked third, emphasizing the role of the thoracolumbar junction. Variance, reflecting the pixel intensity distribution, is also pertinent. This alignment with medical knowledge suggests that the model complements human expertise in radiological assessments. The XGBoost classifier also demonstrated a balanced and clinically meaningful performance, maintaining high sensitivity (AP: 0.93; LAT: 0.73) and strong specificity (AP: 0.73; LAT: 0.94). This balance highlights its capability on how to identify unqualified images while minimizing the misclassification of good QC images. These metrics underscore an important trade-off on determining whether the greater risk in image QC lies in missing unqualified images, which may compromise quality standard, or in unnecessarily rejecting acceptable images, which could increase workload and radiation exposure due to repeated examinations. The optimal threshold between sensitivity and specificity should therefore be guided by institutional priorities, balancing patient safety, diagnostic confidence, and operational efficiency.

The superior performance of the Attention U-Net with the weighted map can be attributed to its synergistic combination of spatial attention mechanisms and adaptive weighting strategies. The attention gates embedded in the network architecture enable selective focus on clinically relevant anatomical structures, such as vertebral bodies, pedicles, and the T12 rib, while suppressing irrelevant background signals and soft-tissue noise. This targeted feature refinement is particularly advantageous in lumbar spine X-rays, where overlapping anatomical structures and variable contrast often obscure key boundaries. The additional incorporation of a weighted map further enhances segmentation accuracy by addressing class imbalance and emphasizing misclassified or underrepresented regions during training. In this study, the weight map assigned higher importance to smaller or more complex structures, such as T12 rib in the AP view, thereby ensuring adequate representation of these regions in the loss optimization process. This dual mechanism forces the network to iteratively refine boundary predictions and improve pixel-level discrimination.

We compared our results with those of Chen et al. [19], who shared similar LSDR classification criteria. Their AP-view analysis included features such as the number of spines, spinal process positions, and pelvic range. Although they used a bilateral shadow of L3 feature not included in our study, we compared the remaining features. As presented in Table 4, our feature set outperformed that of Chen et al., which faced issues such as overexposure, underexposure, and low contrast, leading to lower metrics. Our approach addresses these challenges and achieves higher AUC, ACC, SEN, SPE, and BAC values; additionally, it handles noisy images more effectively.

### 4.2. Limitations

Several limitations should be acknowledged when interpreting the findings of this study. First, the dataset size was relatively limited, which may restrict the generalizability of the model. Although data augmentation was applied to expand the training set, the diversity of anatomical variations, patient body habits, and imaging conditions remain constrained. Larger, multi-center datasets would be needed to validate the robustness of the model across different scanners and populations. Second, we analyzed lumbar rotation in the AP view but struggled to identify its cause, for rotation can result from patient movement during LSDR capture or underlying conditions such as scoliosis. Our limited dataset restricted our ability to distinguish between these causes, which affected the reliability of the model for diagnosing the origins of lumbar rotation. Third, the manual annotation of anatomical landmarks, while performed by experienced specialists, is inherently subjective and time-consuming, introducing potential inter-observer variability. Fourth, all evaluation metrics were derived from internal cross-validation within a single hospital dataset, and performance may not generalize to images acquired using different radiography equipment, imaging protocols, or patient populations at other hospitals. Last, all performance metrics reported in this study are presented as point estimates derived from 5-fold cross-validation. Future research should aim to enhance both the scalability and clinical applicability of the proposed automated QC system by expanding the dataset through multi-center collaborations and incorporating larger, more diverse populations and reducing overfitting, particularly for transformer-based architectures.

## 5. Conclusions

This study introduced an automated QC system that encompasses the complete workflow, from data collection and quality criteria definition to image classification. The proposed framework can serve as a robust template for similar medical applications. The experimental findings demonstrate that the system performed consistently well across various models with XGBoost achieving the highest performance, and these results underscore the reliability and efficacy of the system for QC in medical imaging. Implementing such a system in clinical practice is expected to improve the reliability of LSDR imaging and reduce the manual workload associated with QC. Future research will focus on expanding the ability of the system to diagnose conditions such as vertebral pathologies and enhancing its applicability in clinical settings.

## Figures and Tables

**Table 1:** Mean IoU of segmented model for AP and LAT.

Model	U-Net	Swin-UNet	Attention U-Net	Attention U-Net + Weight Map
AP view	0.816	0.754	0.820	0.832
LAT view	0.884	0.841	0.891	0.915

**Table 2:** Comparison of quality control classification performance across the different models for the anteroposterior (AP) view.

Model	AUC	ACC	SEN	SPE	BAC
MLP	0.7810	0.6978	0.8100	0.5609	0.6854
Attention	0.7599	0.7088	0.7300	0.6829	0.7064
SVM	0.8061	0.7033	0.8100	0.5732	0.6916
RF	0.9112	0.7637	0.9500	0.5365	0.7433
XGBoost	0.9328	0.8406	0.9300	0.7317	0.8309

**Table 3:** Comparison of quality control classification performance across the different models for the lateral (LAT) view.

Model	AUC	ACC	SEN	SPE	BAC
MLP	0.8791	0.8306	0.6727	0.9565	0.8146
Attention	0.8928	0.8468	0.7091	0.9565	0.8328
SVM	0.7156	0.6371	0.1818	1.0	0.5909
RF	0.9071	0.8467	0.7091	0.9565	0.8328
XGBoost	0.8906	0.8468	0.7272	0.9420	0.8347

**Table 4:** Comparison of results with those of Chen et al. study [19].

Model	AUC	ACC	SEN	SPE	BAC
Features in Chen’s	0.585	0.527	0.439	0.6	0.520
Our features	0.9328	0.8406	0.9300	0.7317	0.8309

## Data Availability

The datasets analyzed during the current study are not publicly available due to privacy or institutional restrictions but are available from the corresponding author on reasonable request via email.

## Abbreviations

The following abbreviations are used in this manuscript:

XGBoost	Extreme Gradient Boosting
TP	true positives
TN	true negatives
FP	false positives
FN	false negatives
ACC	accuracy
SEN	sensitivity
SPE	specificity
BAC	balanced accuracy
ROC	receiver operating characteristic
AUC	area under the ROC curve
